# Marital status impacts survival of stage I non-small-cell lung cancer: a propensity-score matching analysis

**DOI:** 10.2144/fsoa-2023-0103

**Published:** 2024-05-15

**Authors:** Li-Hong Qiu, Jia-Qi Song, Feng Jiang, Yuan-Yuan Zhao, Yu'e Liu, Lei-Lei Wu, Guo-Wei Ma

**Affiliations:** 1 Department of Thoracic Surgery, Sun Yat-sen University Cancer Center, State Key Laboratory of Oncology in South China, Collaborative Innovation Center for Cancer Medicine, Guangzhou, 510000, PR China; 2 School of Medicine, Tongji University, Shanghai, 200092, PR China; 3 Department of Oncology, Zhongda Hospital, Southeast University, Nanjing, 210009, PR China

**Keywords:** marital, non-small-cell lung cancer, socioeconomic factors, survival, treatment

## Abstract

**Aim:** This population-based analysis aimed to explore the associations among marital status, prognosis and treatment of stage I non-small-cell lung cancer. **Materials & methods:** The propensity score matching (PSM), logistic regression and Cox proportional hazards model were used in this study. **Results:** A total of 13,937 patients were included. After PSM, 10579 patients were co-insured. The married were more likely to receive surgical treatment compared with the unmarried patients (OR: 1.841, p < 0.001), and patients who underwent surgery also tended to have better survival (HR: 0.293, p < 0.001). **Conclusion:** Compared with unmarried patients, a married group with stage I NSCLC had timely treatment and more satisfactory survival. This study highlights the importance of prompt help and care for unmarried patients.

## Abstract (plain language summary)

Marital status had an effect on survival of early-stage NSCLC patients.

Married patients were more likely to receive surgical treatment compared with the unmarried patients.

Socioeconomic factors of patients should be paid attention.

This study highlights the importance of prompt help and cares for unmarried stage I NSCLC patients.

Lung cancer still ranks first in the global cancer spectrum of morbidity and mortality [1]. In lung cancer patients, non-small-cell lung cancer (NSCLC) is the most common pathological type, accounting for about 80%–85%, and most of them are advanced when diagnosed, with a 5-year survival rate of about 5% [2]. Thus, early diagnosis is very important for NSCLC patients, and surgery has been considered the best curative option for stage I NSCLC. However, it is still reported that a third of patients die of tumor recurrence [3]. Therefore, it frequently makes sense to precisely estimate the risk associated with patient's prognosis for survival and to regularly check on patients who are at high risk.

Several factors affect the prognosis of lung cancer. Most studies to date have focused on patient clinical information, pathological features and gene types [^4-7^]. To our knowledge, the association between marital status and survival, and treatment remains unclear. Previous studies suggested that among individuals with malignant tumors, including penile [8,9], testicular [10], colon [11] and breast cancers [12,13], married individuals had better prognoses than single, separated, divorced and widowed patients. In this study, therefore, we aimed to investigate the association of marital status with treatment and survival in patients with stage I NSCLC.

## Materials & methods

### Data sources

The data of patients diagnosed with stage I NSCLC from 2010 to 2015 was extracted from the Surveillance, Epidemiology and End Results (SEER) database. The data were downloaded using SEER*STAT software (version 8.3.9). The SEER program of the National Cancer Institute in the USA collects data from 18 population-based registered cancer institutes, covering approximately 28% of cancer cases in USA.

### Patients

**Figure 1.**
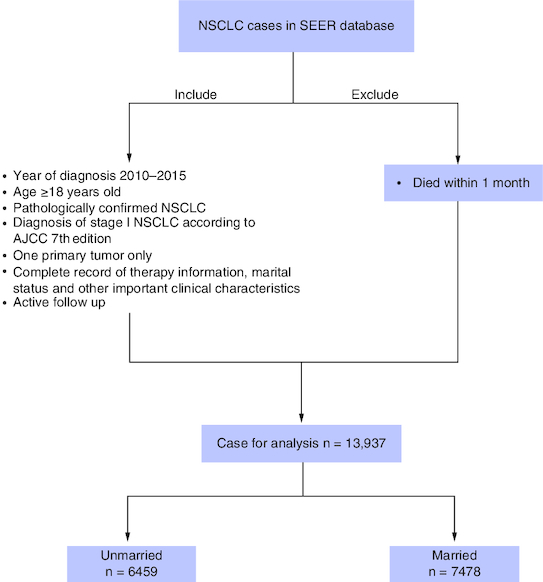
The diagram of this study. AJCC: American Joint Committee on Cancer; NSCLC: Non-small-cell lung cancer; SEER: Surveillance, Epidemiology and End Results.

The ethics committee of our cancer center approved this study and considered this study exempt from ethical review because existing data without patient identifiers were used. This study included patients who were histologically diagnosed with NSCLC as their first primary malignancy from January 2010 to December 2015 (primary site: C34.0–C34.9). Patients who met the following criteria were enrolled in the study: age older than 18 years; pathologically confirmed NSCLC (histologic types were selected as adenocarcinoma [codes: 8140, 8250–8255, 8260, 8310, 8323, 8333, 8480, 8481, 8490, 8550, 8570, 8574], squamous cell carcinoma [codes: 8052, 8070–8074, 8083, 8084] and other NSCLC [codes: 8012, 8013, 8022, 8031–8033, 8035, 8046, 8050, 8082, 8123, 8200, 8201, 8430, 8560, 8980]; diagnosis of stage I NSCLC according to American Joint Committee on Cancer (AJCC) 8th Edition in 2010–2015; one primary malignant lung tumor only; complete record of therapy information, marital status and other critical clinical characteristics; actively followed ups. Patients who did not meet the study's registration requirements or who died within a month were major exclusion factors. Given that patients might die of perioperative complications who die within one month from diagnosis, we excluded those patients. All patients were divided into married and unmarried groups according to their marital status, and the unmarried group included divorced, separated, single (never married) and widowed. Details of the patient selection process are shown in Figure 1.

### Treatment

According to the SEER database, the primary treatment approaches for stage I NSCLCs included surgery, chemotherapy and radiotherapy (beam radiation, combination of beam with implants or isotopes, radiation source not specified, radioactive implants). Several surgical approaches were recorded, such as lobectomy, sublobectomy (segmental resection and wedge resection) and pneumonectomy.

### Follow-up

The included patients had a clear survival time and survival status. Cancer-specific survival (CSS), i.e., the duration from the date of diagnosis to death caused by lung cancer, was regarded as our observational end point. The follow-up information on the SEER database was updated in November 2021. The median follow-up time was 48.0 months (range 1.0–107.0 months).

### Statistical analysis

Statistical analysis was performed using SPSS statistics 25.0 software (IBM SPSS, Inc, IL, USA), and GraphPad Prism 8 (https://www.graphpad.com/scientific-software/prism/). Pearson's χ^2^ statistic, Student's *t*-test and logistic regression analysis were used to determine the associations between marital status and other clinical features. Propensity score matching (PSM) with 0.001 matching tolerance was used to balance baseline characteristics between married and unmarried groups. Univariable and multivariable proportional hazards regression model were used to calculate the hazard ratio (HR) and 95% CI and assess the effect of marital status and other clinical characteristics, on CCS. Kaplan–Meier analysis and log-rank tests were performed to compare survival curves between the groups. Statistical tests were considered statistically significant with two-sided p-value <0.05.

## Results

### Patient characteristics

The clinical characteristics of the patients are presented in Table 1. A total of 13,937 patients fulfilled our inclusion criteria and were included in the study population. Among them, 6459 (46.3%) patients were unmarried and 7478 (53.7%) were married. Overall, significant differences in all clinicopathological variables except laterality were observed between the groups classified according to marital status (Table 1, all p < 0.05) before PSM. To better compare the two groups, using PSM at a ratio of 1:1, 10,579 patients remained in the group. Except for race (p < 0.001) and pathology (p = 0.012), there were no significant differences in the clinicopathological patient characteristics between the unmarried and married groups after PSM, as shown in Table 1.

**Table 1.** The baseline clinical characteristics of enrolled patients with stage I non-small-cell lung cancer before and after propensity-score matching.

Characteristic	Before propensity score matching			After propensity score matching		
	Unmarried n = 6459 (%)	Married n = 7478 (%)	p-value	Unmarried n = 5289 (%)	Married n = 5290 (%)	p-value
Age	70 ± 10.3	69 ± 10.2	<0.001	69 ± 10.3	69 ± 9.6	0.373
– ≤70	3258 (50.4)	4251 (56.8)		2957 (55.9)	3011 (56.9)	
– >70	3201 (49.6)	3227 (43.2)		2332 (44.1)	2279 (43.1)	
Sex			<0.001			0.780
– Female	4375 (67.7)	3477 (46.4)		3228 (61.0)	3243 (61.3)	
– Male	2084 (32.3)	4001 (53.6)		2061 (39.0)	2047 (38.7)	
Race			<0.001			<0.001
– White	5188 (80.4)	6283 (83.9)		4200 (79.4)	4425 (83.6)	
– Black	945 (14.6)	523 (7.0)		787 (14.9)	386 (7.3)	
– Other	326 (5.0)	672 (9.1)		302 (5.7)	479 (9.1)	
Laterality			0.616			0.937
– Left	2633 (40.8)	3017 (40.3)		2155 (40.7)	2160 (40.8)	
– Right	3826 (59.2)	4461 (59.7)		3134 (59.3)	3130 (59.2)	
Histology			<0.001			0.012
– Adenocarcinoma	3897 (60.2)	4745 (63.4)		3335 (63.1)	3386 (64.1)	
– Squamous	1754 (27.3)	1818 (24.3)		1350 (25.5)	1235 (23.3)	
– Other	808 (12.5)	915 (12.3)		604 (11.4)	669 (12.6)	
Grade			<0.001			0.114
– I	1024 (15.9)	1432 (19.1)		946 (17.9)	1006 (19.1)	
– II	2404 (37.2)	3048 (40.8)		2142 (40.5)	2071 (39.1)	
– III	1558 (24.1)	1725 (23.1)		1246 (23.6)	1201 (22.7)	
– IV	55 (0.9)	51 (0.7)		47 (0.9)	37 (0.7)	
– Unknown	1418 (21.9)	1222 (16.3)		908 (17.2)	975 (18.4)	
Stage			0.001			0.753
– IA	5472 (84.7)	6181 (82.7)		4425 (83.7)	4413 (83.4)	
– IB	987 (15.3)	1297 (17.3)		864 (16.3)	877 (16.6)	
Surgery			<0.001			0.775
– No	2240 (34.8)	1673 (22.4)		1420 (26.8)	1407 (26.6)	
– Yes	4219 (65.2)	5805 (77.6)		3869 (73.2)	3883 (73.4)	
Chemotherapy			0.022			0.317
– No	6162 (95.5)	7070 (94.5)		5047 (95.4)	5025 (95.0)	
– Yes	297 (4.5)	408 (5.5)		242 (4.6)	265 (5.0)	
Radiation			<0.001			0.675
– No	4698 (72.7)	6029 (80.6)		4094 (77.4)	4113 (77.8)	
– Yes	1761 (27.3)	1449 (19.4)		1195 (22.6)	1177 (22.2)	
Tumor size	20 ± 6.3	20 ± 6.3	0.025	20 ± 6.3	20 ± 6.2	0.669

### Univariable & multivariable analyses

**Figure 2.**
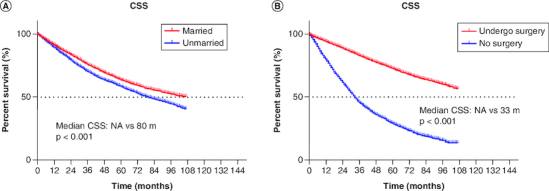
Cancer-specific survival curve for cohort of patients with non-small-cell lung cancer according to the status. **(A)** Marriage and **(B)** surgery after propensity-score matching. CSS: Cancer-specific survival.

Univariable and multivariable Cox regression analyses were performed to determine the correlations between clinical characteristics and CSS. As shown in Table 2, univariable and multivariable analyses identified the following clinical characteristics as significant CSS prognostic factors in all patients: sex, age, differentiation, surgery, chemotherapy, radiotherapy, tumor size and marital status. The results showed that marital status could be an independent prognostic factor in stage I patient (HR: 0.811 95% CI: 0.763–0.861, p < 0.001). In addition, married and underwent surgery had better CSS (Figure 2).

**Table 2.** Univariable and multivariable Cox regression analysis for cancer-specific survival.

Characteristics	Univariable analysis			Multivariable analysis		
	HR	95% CI	p-value	HR	95% CI	p-value
Sex (female vs male)	1.828	1.722–1.940	<0.001	1.439	1.353–1.530	<0.001
Age (≤70 vs >70)	1.951	1.838–2.072	<0.001	1.541	1.448–1.641	<0.001
**Race**						
– White	1	reference		1	reference	
– Black	0.932	0.847–1.026	0.152	0.941	0.854–1.037	0.218
– Other	0.610	0.533–0.698	<0.001	1.541	1.448–1.641	<0.001
Laterality (right vs left)	0.929	0.874–0.986	0.016	0.971	0.914–1.032	0.342
**Histology**						
– Adenocarcinoma	1	reference		1	reference	
– Squamous	1.907	1.786–2.035	<0.001	1.411	1.319–1.509	<0.001
– Other	1.12	1.016–1.235	0.023	0.957	0.867–1.058	0.391
Differentiation (Grade I vs II vs III vs IV)	1.336	1.308–1.365	<0.001	1.070	1.044–1.097	<0.001
Stage (IA vs IB)	0.936	0.863–1.015	0.110			
Surgery (no vs yes)	0.261	0.245–0.277	<0.001	0.293	0.263–0.325	<0.001
Chemotherapy (no vs yes)	1.538	1.362–1.737	<0.001	1.311	1.159–1.482	<0.001
Radiotherapy (no vs yes)	2.898	2.723–3.085	<0.001	0.744	0.673–0.822	<0.001
Marital status (unmarried vs married)	0.810	0.763–0.860	<0.001	0.811	0.763–0.861	<0.001
Tumor size (cm)	1.033	1.028–1.038	<0.001	1.019	1.014–1.024	<0.001

### Associations between marital status & treatment

**Figure 3.**
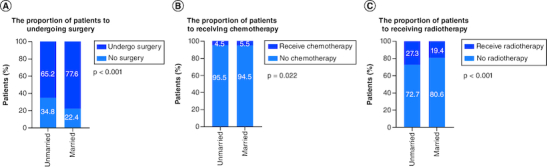
The proportion of the patients with non-small-cell lung cancer. In **(A)** surgery, **(B)** chemotherapy and **(C)** radiotherapy according to marital status.

**Figure 4.**
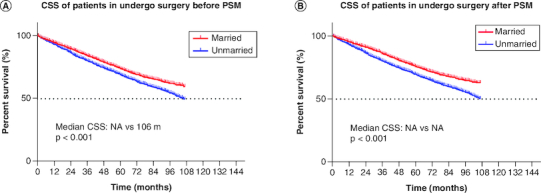
Kaplan–Meier survival curves. **(A)** CSS in undergo surgery patients before PSM, **(B)** CSS in undergo surgery patients after PSM. CSS: Cancer-specific survival; PSM: Propensity score matching.

The majority of the patients in married cohorts received surgery (unmarried vs married: 65.2 vs 77.6%, p < 0.001, Figure 3 & Table 3). In addition, married patients (5.5%) and unmarried patients (4.5%) had similar odds of receiving chemotherapy (Figure 3 & Table 3). As for radiotherapy, 27.3% of unmarried patients and 19.4% of married patients had received radiotherapy (Figure 3 & Table 3). In addition, we further stratified the association between therapeutic and marital status. The study showed that among all patients who underwent surgery, both before (median CSS:NA vs 106 months, p < 0.001) and after (p < 0.001) PSM, married group had better CSS than unmarried group. Considering that the married group had a better CSS, surgery was optimal for all married patients (Figure 4).

**Table 3.** Multivariable Logistic regression analysis for different treatment according to marital status (method is enter selection).

Characteristics	Surgery			Chemotherapy			Radiotherapy		
	OR	95% CI	p-value	OR	95% CI	p-value	OR	95% CI	p-value
Sex (female vs male)	0.777	0.717–0.841	<0.001	1.118	0.957–1.306	0.16	1.197	1.101–1.302	<0.001
Age (≤70 vs >70)	0.369	0.341–0.399	<0.001	0.549	0.467–0.645	<0.001	2.317	2.133–2.517	<0.001
Race									
– White	1	reference		1	reference		1	reference	
– Black	0.812	0.718–0.918	0.001	1.106	0.872–1.403	0.406	1.009	0.84–1.152	0.897
– Other	1.467	1.243–1.731	<0.001	0.947	0.702–1.277	0.719	0.519	0.43–0.628	<0.001
Laterality (right vs left)	1.093	1.010–1.182	0.026	1.022	0.875–1.194	0.784	0.895	0.825–0.972	0.008
Marital status (unmarried vs married)	1.841	1.698–1.995	<0.001	1.163	0.992–1.364	0.063	0.654	0.601–0.712	<0.001
Tumor size (cm)	0.966	0.960–0.972	<0.001	1.065	1.051–1.078	<0.001	1.026	1.020–1.033	<0.001

## Discussion

With advances in medicine, increasing research has focused on molecular mechanisms, clinical information, pathological features and other factors when exploring the prognosis of patients with lung cancer [^14-16^]. Results from studies focusing on socioeconomic factors suggest that neighborhood deprivation, caregiver burden, marital status, insurance status and others may affect the prognosis of patients with malignant tumors [^17-23^]. The present study analyzed the association of marital status with stage at diagnosis, treatment and survival among patients diagnosed with NSCLC from 2010 to 2015 in the SEER database. A total of 13,937 patients with stage I NSCLC were included in this study, and their clinical and socioeconomic features were investigated. Our findings showed that that the married group had better CSS than the unmarried patients in all populations (Figure 2A). Further studies showed that more married patients chose surgery (Figure 3), and they had better CSS among all surgical patients (Figure 4).

The results of our study showed that treatment and CSS varied with different marital statuses, which was in accordance with the findings of Chen *et al.* for NSCLC [23]. Patients who were married, single, separated, divorced, and widowed were included. We grouped the patients into married and unmarried (single, separated, divorced, widowed). The univariable analysis showed that unmarried patients had worse survival outcomes, which was in accordance with some studies involving other malignant tumors [^24-28^]. Several possible mechanisms may explain the relation between marital status and survival outcomes. First, for stage I NSCLC patients, at a time when surgery is still the best option, given that married patients are more likely to receive support from family members, they may be inclined to aggressive treatment (Figure 3). When before or after treatment including surgery, chemotherapy and radiotherapy, and daily life and work are therefore affected, most married patients can recover faster due to the company and careful care of their families, leading to a satisfactory survival rate. At the same time, this study also observed that a strong positive correlation exists between marital status and early diagnosis, and surgery (Table 3), of which the two factors were essential for satisfactory outcomes for patients with NSCLC (Table 2 & Figure 2).

Secondly, besides physical care, mental support is urgently needed for patients with cancer, which is largely dependent on the stable and comforting relationship of marriage. Married patients often receive help from their spouse throughout the course of diagnosis and treatment. However, unpleasant and upsetting relationships generate depression, leading to divorce or separation, which could account for poor prognosis [8,9]. Satin *et al.* reported that depression could act as a predictor for disease progression and motility of malignant tumor by a meta-analysis [29]. Without support from their spouses, unmarried patients are prone to suffer from more emotional pressure, as well as worse socioeconomic situations, which may be associated with poor prognosis among patients with NSCLC.

Previous work from Huang *et al.* also found that marital status had an impact on the survival of stage IA NSCLC patients [24]. Compared with their study, we first defined married and unmarried patients and screened out suitable groups. Considering that society has different views on marriage at different times, we only selected stage I NSCLC patients recorded in the SEER database from 2010 to 2015. Secondly, we used PSM to make the results more convincing. In addition, we further stratified the effects of treatment modalities (such as surgery) on patients with different marital statuses. Finally, our findings suggested that a married group with stage I NSCLC had timely treatment and more satisfactory survival compared with unmarried patients. This study highlights the importance of prompt help and care for unmarried patients.

This study has several limitations. We did not categorize the precise treatment approach, as well as the cost for every treatment. In the meantime, as the SEER database only records whether patients have received chemotherapy or not, it is temporarily impossible to obtain the chemotherapy regimen used by patients and when they have used chemotherapy. We were also unable to observe the dynamic changes in marital status after diagnosis and treatment because the SEER database is retrospective. The adoption of PSM in this study balances baseline patient characteristics between groups. Moreover, we were unable to determine the physiological and psychological changes in patients according to their marital status, which could have provided more insights into the association between prognosis and marital status. Therefore, more studies are necessary to further explore the effect of marital status on diagnosis, treatment, and prognosis of patients with stage I NSCLC. Finally, the married patients had a survival advantage, but this does not mean that they could deny receiving treatment.

## Conclusion

In conclusion, this study shows the present study showed that marriage was independent protective prognostic factors in stage I NSCLC. The results based on SEER database analysis are more likely to reveal that these married patients may be more inclined to choose surgical treatment to achieve timely treatment and a more satisfactory survival time. Therefore, it is recommended that doctors pay close attention to the marital status of patients and provide psychological or other support for unmarried patients in time.
